# Aids to Medicine, Part No. 1. The General Diseases: Diseases of the Lungs, Heart, Blood Vessels and Liver

**Published:** 1883-06

**Authors:** 


**Aids to Medicine. Part No. 1. The General Diseases: Diseases of the Lungs, Heart, Blood Vessels and Liver.** By C. E. ARMAND SEMPLE, B. A., M. D., etc. New York: G. P. Putnam's Sons, 27 West Twenty-third street.

This is a little book which may prove useful as an introduction to the study of medicine, or as a refresher to the memory for those going up for examination. It is one of the "Students' Aid Series," and is handsomely printed in 16mo. form, convenient for the pocket. Price 35c.

